# How Native Background Affects Human Performance in Real-World Visual Object Detection: An Event-Related Potential Study

**DOI:** 10.3389/fnins.2021.665084

**Published:** 2021-04-30

**Authors:** Yue Wang, Jianpu Yan, Zhongliang Yin, Shenghan Ren, Minghao Dong, Changli Zheng, Wei Zhang, Jimin Liang

**Affiliations:** ^1^School of Electronic Engineering, Xidian University, Xi'an, China; ^2^School of Life Science and Technology, Xidian University, Xi'an, China; ^3^Southwest China Research Institute of Electronic Equipment, Chengdu, China

**Keywords:** visual processing, object detection, native background, scene complexity, attentional state, event-related potential

## Abstract

Visual processing refers to the process of perceiving, analyzing, synthesizing, manipulating, transforming, and thinking of visual objects. It is modulated by both stimulus-driven and goal-directed factors and manifested in neural activities that extend from visual cortex to high-level cognitive areas. Extensive body of studies have investigated the neural mechanisms of visual object processing using synthetic or curated visual stimuli. However, synthetic or curated images generally do not accurately reflect the semantic links between objects and their backgrounds, and previous studies have not provided answers to the question of how the native background affects visual target detection. The current study bridged this gap by constructing a stimulus set of natural scenes with two levels of complexity and modulating participants' attention to actively or passively attend to the background contents. Behaviorally, the decision time was elongated when the background was complex or when the participants' attention was distracted from the detection task, and the object detection accuracy was decreased when the background was complex. The results of event-related potentials (ERP) analysis explicated the effects of scene complexity and attentional state on the brain responses in occipital and centro-parietal areas, which were suggested to be associated with varied attentional cueing and sensory evidence accumulation effects in different experimental conditions. Our results implied that efficient visual processing of real-world objects may involve a competition process between context and distractors that co-exist in the native background, and extensive attentional cues and fine-grained but semantically irrelevant scene information were perhaps detrimental to real-world object detection.

## 1. Introduction

Natural scenes are highly complex and diverse, containing a massive variety of visual information that would require an unrealistic amount of cognitive resources to be effectively processed (Zhang et al., 2016). We humans benefit from sophisticated attention mechanisms that are modulated by bottom-up stimulus-driven and top-down goal-directed factors (Yantis, 2000) and help us select and process vital information to ensure the functioning of higher-level mental processes (Wolfe, 2003). Top-down and bottom-up attentions, including working memory, competitive selection, top-down sensitivity control, and bottom-up salience filters (Knudsen, 2007), jointly create a biased representation of the external visual world according to both visual salience (i.e., target-background contrast) and task relevance (i.e., attended or unattended) (Spragure et al., 2018). As task difficulty increases, such as to distinguish between objects with similar features (Boudreau et al., 2006) or with task-irrelevant disturbances (Desimone and Duncan, 1995), higher quality of encoded information is required, and a corresponding enhancement of partial neural responses may be produced (Painter et al., 2014).

Visual objects processing relies on the neural encoding of visual information from both target and background (Luo and Ding, 2020). The background surrounding a visual object has a significant impact on target detection and identification performance (Naber et al., 2012), even more so than the semantic category of the object itself (Hagen and Laeng, 2016). The background seems to be a double-edged sword for visual object processing. On the one side, background context can promote the effective processing of visual objects (Sun et al., 2011; Barenholtz, 2014) and speed up the behavioral responses by guiding human attention oriented voluntarily to spatial position or visual features (Treue and Trujillo, 1999). On the flip side, the visual background may be accompanied by task-irrelevant interferences, which often inevitably reduces the task performance (Theeuwes, 2004). Specifically, in visual target detection, the presence of a salient task-irrelevant background increased the false alarm rate and slowed down the speed for decision-making (Luo and Ding, 2020). In visual target classification, the performance for classify objects embedded in complex backgrounds (especially in semantically inconsistent backgrounds) was significantly lower than that for isolated objects (Davenport and Potter, 2004), which may be attributed to the increased difficulty of target-background splitting in complex scenes (Torralbo and Beck, 2008; Prass et al., 2013).

Most previous studies have used synthetic or curated visual stimuli to investigate the top-down and bottom-up attentions in visual processing, such as embedding natural (Sun et al., 2011; Prass et al., 2013; Hagen and Laeng, 2016) or artificial (Smout et al., 2019; Luo and Ding, 2020) objects into different natural or artificial scenes, or adjusting images to various resolutions (Torralba, 2009; Barenholtz, 2014). Although these synthetic and curated stimuli can introduce a controlled amount of variation or difficulty to the task and allow the investigation of neural coding in relation to these factors (Cadieu et al., 2014), they are difficult to expose or maintain the contextual effects in real world, thus inevitably strips away the effects of some unknown but crucial factors in the native background. For example, the “animate monitoring” hypothesis (New et al., 2007) could be reversed due to the adjustment of the surrounding scenes (Hagen and Laeng, 2016). We argue that the coherent contextual information is essential for studying the real-world object processing problems.

The current study investigated the effects of native background on visual object detection through a vehicle detection task under different scene complexity and background task-relevance conditions. In our experiments, visual stimuli with various background contents such as woods, buildings, roads, parking lots, construction sites, or fields were collected from the VEDAI dataset (Razakarivony and Jurie, 2016). The scene complexity was defined by a combination of qualitative and quantitative measures. The presentation condition of simple scene was compared with that of complex scene. Moreover, the task-relevance of visual background was controlled by an attention-guiding strategy. The condition in which participants fully focused on the vehicle detection task (single-task) was compared with condition in which participants should also actively observe the background details while performing the vehicle detection task (dual-task). Brain responses were recorded using electroencephalogram (EEG) while the participants undertook the tasks.

In visual object detection, all content within the visual field competes for processing resources, with distractors in the background inevitably attracting bottom-up attention and competing with objects for neural representations (Cave and Chen, 2016), and top-down attention provides a bias signal to resolve competing interactions between object and distractors in the visual cortex according to task requirements (Scalf et al., 2013). We speculate that object detection in native background may also involve competition between different attributes in the background, and this competition can be biased by both bottom-up and top-down attentional mechanisms. We expect to see distinct brain response dynamics in scenes with varied scene complexity or with different attention states (attend or unattended) to the background.

## 2. Materials and Methods

### 2.1. Participants

Fifty healthy individuals (26 males and 24 females) from Xidian University [age range: 21–31 years, mean = 23.98, standard deviation (SD) = 1.82] participated in this study. All participants were right-handed, had normal or corrected-to-normal vision, and had no history of psychiatric or neurological disorders. Two participants (one male and one female) were discarded in the ERP analysis because of excessive noise in EEG data. Consequently, the EEG data from a total of 48 participants were analyzed. All participants provided written informed consent prior to the start of the experiment and received monetary payment. The experimental procedures were conducted in accordance with the Helsinki Declaration of 1975, as revised in 2000.

### 2.2. Stimuli

**Figure 1:**
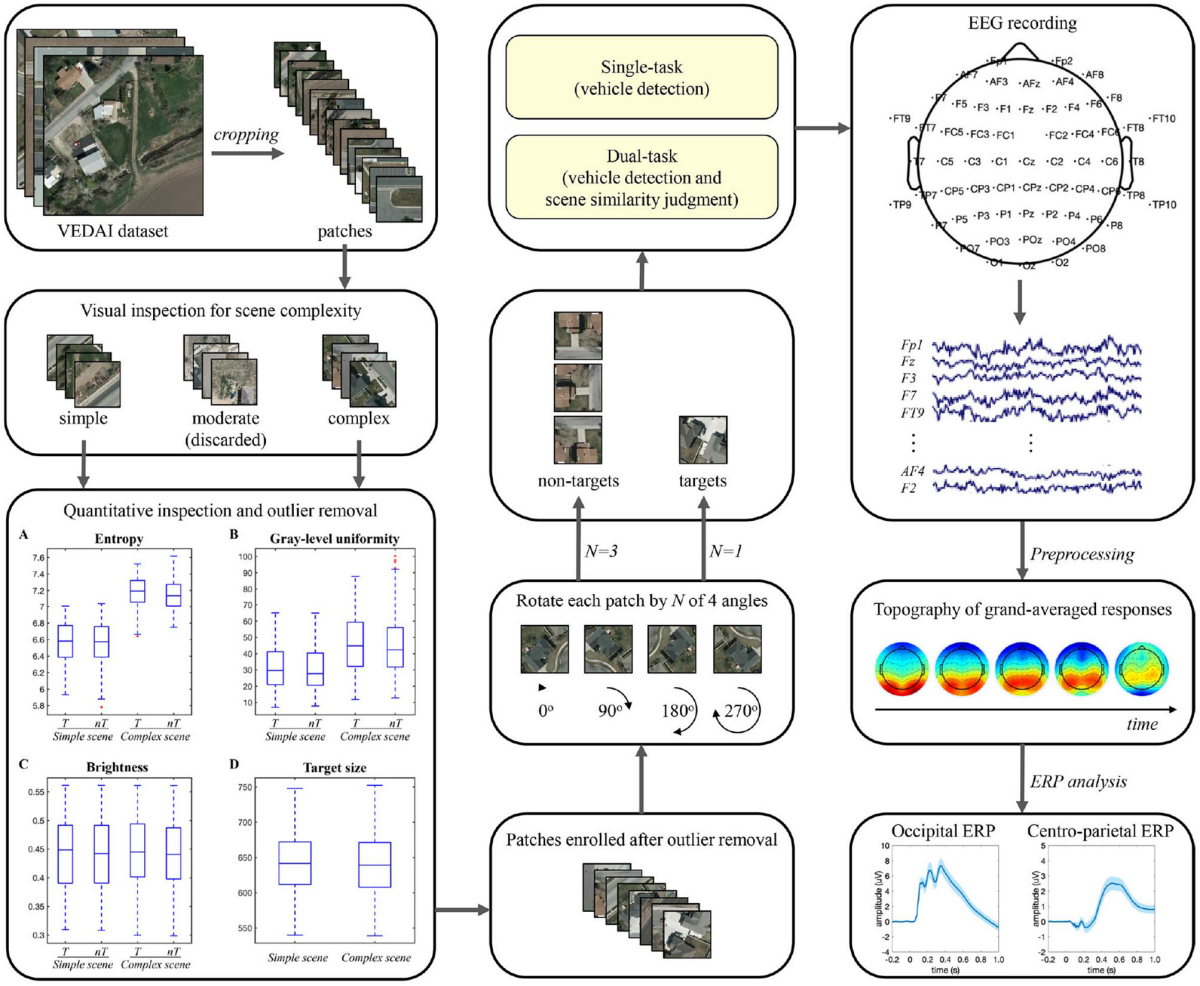
Pipeline of stimulus set generation and EEG data analysis.

In the experiment, stimuli were presented on a 23-inch liquid crystal display monitor with a resolution of 1960 × 1080 pixel^2^ and a refresh rate of 60 Hz, using the Psychophysics Toolbox (version 3) presentation software (http://www.psychtoolbox.org) under MATLAB (version 18a). Participants were seated in a comfortable armchair in an electrically shielded laboratory with a viewing distance of approximately 70 cm. The pipeline of stimulus set generation and data analysis in this study is illustrated in Figure 1.

Stimuli for vehicle detection were collected from the VEDAI dataset (Razakarivony and Jurie, 2016) in the publicly available Utah AGRC database (http://gis.utah.gov/). Only the large-size (1,024 × 1,024 pixel^2^) aerial ortho-normalized color images were used in this study. We manually cropped 2,416 patches from the color images (1,208 target patches, each containing only one vehicle and of similar size across patches, and 1,208 non-target patches) and then resampled the patch size to 240 × 240 pixel^2^. Considering that these patches were designed to be viewed by human subjects, a visual check of scene complexity was first performed based on the type, number, distribution, and similarity of background items. Patches with high confidence were directly assigned to the simple or complex group, and other indistinguishable patches were assigned to the moderate group. Although the moderate patches could be included in either the simple or complex group using the subsequent quantitative measures, they may reduce the intergroup differences in brain response signals associated with scene complexity and make subsequent analysis challenging. Therefore, the moderate group (29.5% of the total patches) was excluded from the experiment. Thereafter, the remaining patches were further evaluated quantitatively in terms of scene complexity, brightness, and vehicle size. The scene complexity was assessed by the information entropy and gray-scale uniformity indices (Peters and Strickland, 1990), the brightness was characterized by the mean value of the intensity channel in HSI space, and the size of each vehicle was calculated based on the four-point coordinates of its manually-labeled location marker. Outliers exceeding 1.5 times the interquartile range of information entropy, gray-scale uniformity, brightness, or vehicle size measures were sequentially removed from the stimulus set. Since the removal of outliers based on one measure may result in new outliers for other measures, the outliers' removal process was iterated many times. Finally, 17.5% of the total candidate patches were removed, and an equal number of patches of four types were retained.

The above screening procedure eventually yielded 1,280 patches, including 320 patches of each of the four types: simple scene with vehicle (simple-scene target), simple scene without vehicle (simple-scene non-target), complex scene with vehicle (complex-scene target), and complex scene without vehicle (complex-scene non-target). The simple-scene and complex-scene stimulus groups showed significant difference in scene complexity, as measured by the indices of information entropy (*p* < 0.001, two-sample *t*-test) and gray-level uniformity (*p* < 0.001, two-sample *t*-test). There was no significant difference in brightness (*p* = 0.553, two-sample *t*-test) or vehicle size (*p* = 0.478, two-sample *t*-test) between the two groups (Table 1). Moreover, there was no significant difference in information entropy (target vs. non-target: 6.87 ± 0.38 vs. 6.85 ± 0.37, mean ± SD, *p* = 0.253, two-sample *t*-test), gray-level uniformity (target vs. non-target: 39.09 ± 17.53 vs. 38.18 ± 17.76, mean ± SD, *p* = 0.356, two-sample *t*-test), and brightness (target vs. non-target: 0.443 ± 0.067 vs. 0.440 ± 0.065, mean ± SD, *p* = 0.383, two-sample *t*-test) between target and non-target patches.

**Table 1:** Quantitative indicators of stimulus patches.

	**Simple scene**	**Complex scene**	***p*-value**
InfoEntropy	6.55 ± 0.26	7.16 ± 0.18	<0.001
Uniform	31.09 ± 13.34	45.98 ± 18.25	<0.001
Luminance	0.44 ± 0.07	0.44 ± 0.07	0.553
Vehicle size	642.31 ± 45.10	639.83 ± 43.30	0.478

*InfoEntropy, information entropy; Uniform, gray-scale uniformity. Values in the table are expressed as mean ± SD. The p-values were obtained with two-tailed two-sample t-test*.

**Figure 2:**
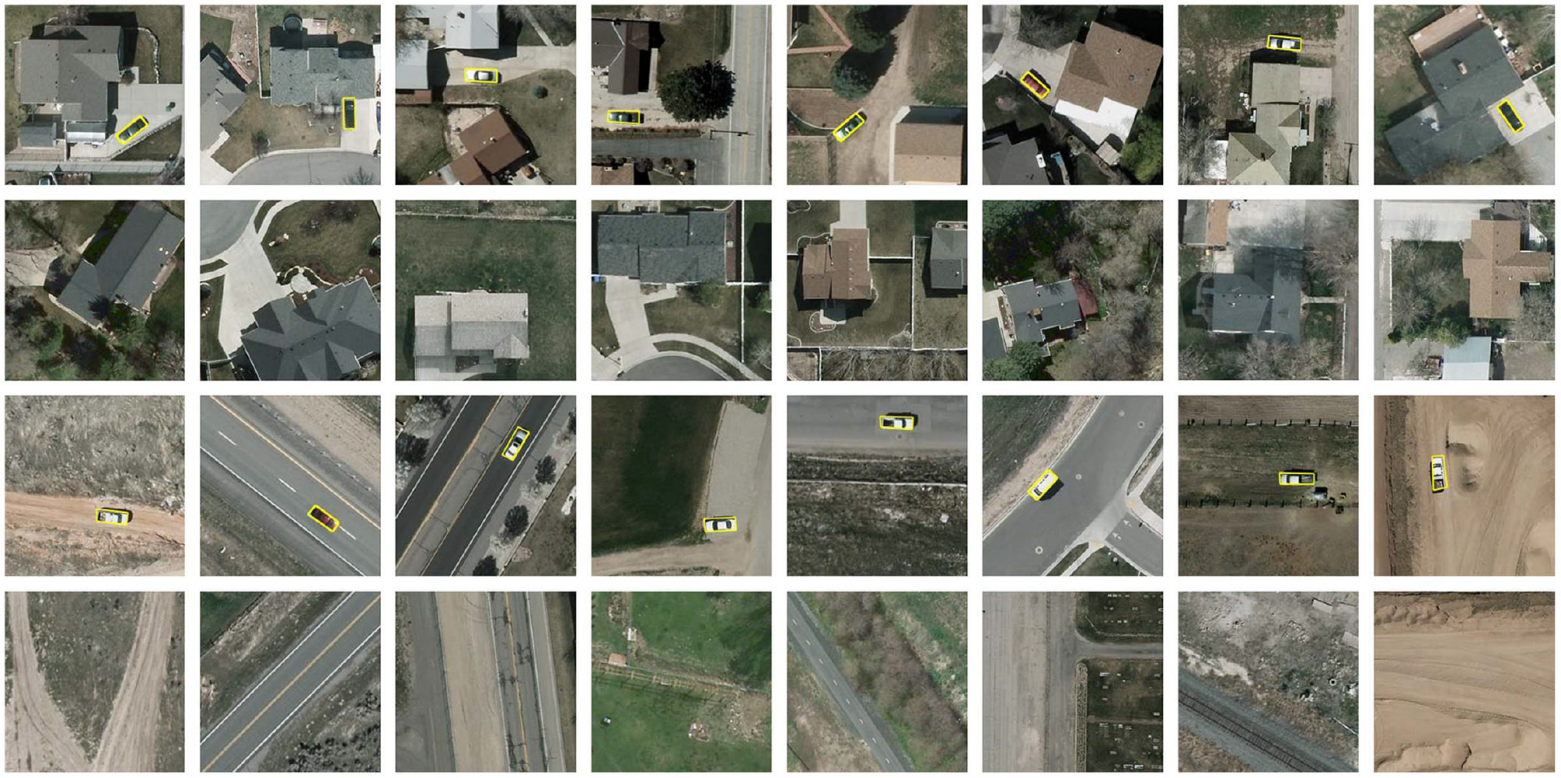
Stimuli for vehicle detection. From top to bottom each row: complex-scene target, complex-scene non-target, simple-scene target and simple-scene non-target. Vehicles are marked with yellow boxes.

Some examples of stimuli are shown in Figure 2. The stimulus set for practicing was constructed by randomly selecting 20 patches from each type and randomly rotating each patch by 0, 90, 180, or 270 degrees once. In the testing phase, we employed the oddball paradigm commonly used in previous target detection studies (O'Connell et al., 2012; Luo and Ding, 2020) and constructed the stimulus set for testing using one random rotation of the remaining target patches and three random rotations of the remaining non-target patches, so the ratio of the number of target to non-target patches was 1:3 (600:1800).

### 2.3. Experimental Design

**Figure 3:**
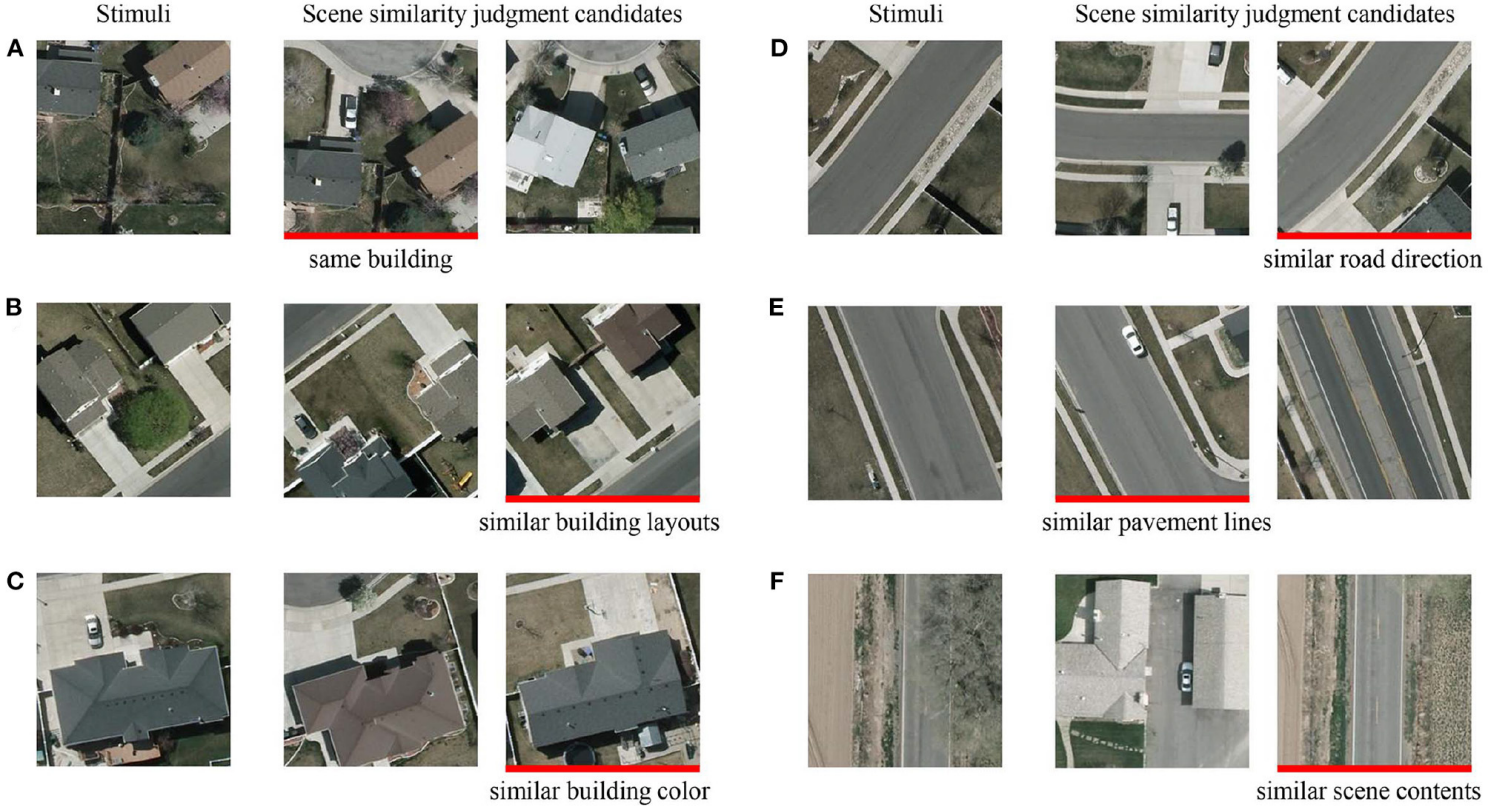
Examples of the two-alternative forced-choice scene similarity judgment questions. Red markers under the candidates indicate that their scenes were more similar to that of their corresponding stimuli due to the presence of the same building **(A)**, similar building layouts **(B)**, similar building color **(C)**, similar road direction **(D)**, similar pavement lines **(E)**, or similar scene contents **(F)**.

The experiment consisted two tasks: (1) the single-task, in which the participants were fully focused on vehicle detection and made keystroke judgments about whether the stimulus contained a vehicle or not, and (2) the dual-task, in which the participants should actively observe the background scenes while performing the vehicle detection task. In the dual-task, in order for participants to actively attend to the background details, all practicing stimuli and one-third of the testing stimuli (50 for each type, for the sake of experiment time) were followed by a two-choice question on scene similarity. For each question, two candidate patches with the same resolution as the stimuli were presented side-by-side as illustrated in Figure 3. Participants were informed that the presence or absence of a vehicle should not be taken into account in judging the similarity of the scenes. All candidate patches were also manually cropped from the VEDAI color images. One of the candidate patches has a scene more similar to that of the previous stimulus, such as with the same buildings (Figure 3A), similar building layouts (Figure 3B) or building color (Figure 3C), similar road direction (Figure 3D) or pavement lines (Figure 3E), or similar scene contents (Figure 3F). Candidate patches with similar scenes appeared randomly at the left or right position, and participants were asked to press a key to answer which candidate patch was more similar to the previous stimulus.

For both single and dual tasks, prior to the testing phases, participants were cued to perform a practicing block to adapt to the experimental environment and key pressing operations. During the single-task practicing, a black crosshair was firstly displayed in the center of screen for 2 s to remind participants that the experiment was about to begin. Then, a stimulus for vehicle detection was presented centrally until the participants pressed a judgement key, and a feedback image would appear on the screen for 1 s. If the stimulus contained a vehicle, the vehicle would be marked with a yellow box in the feedback image; otherwise, the feedback image was the same as the stimulus. During the dual-task practicing, the presentation of stimuli and feedback images was the same as that in the single-task, then a black crosshair inter-stimulus interval (ISI) was presented for 1.25–1.5 s followed by a 1-s red crosshair informing that the scene similarity judgment question was about to appear. More importantly, since participants needed to make lateral eye movements when observing candidates for the scene similarity questions, the red crosshair before the question could guide participants' attention to focus on the center of the screen, thus avoided the unnecessary eye movements that would affect the quality of brain response signals. After the participants finished the scene similarity judgment, a feedback would appear on the screen for 1 s with the incorrect option being obscured and the basis for judgement being presented in text form below the correct option.

**Figure 4:**
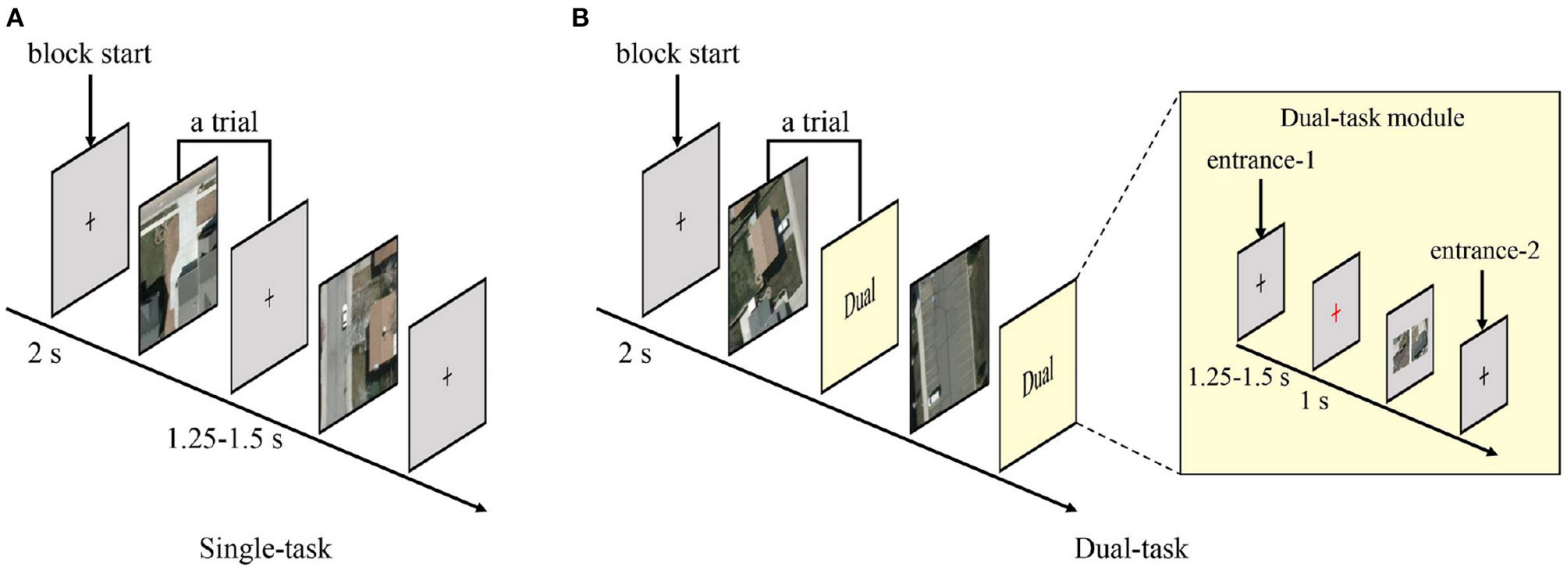
Temporal structures of stimulus presentation. **(A)** Paradigm of the single-task. Each trial consisted of a vehicle detection stimulus and an inter-stimulus interval. **(B)** Paradigm of the dual-task. Each trial consisted of a vehicle detection stimulus and a dual-task module. One-third trials entered the dual-task module from entrance-1 and the remaining trials from entrance-2.

The temporal structure of testing blocks were similar with that of practicing blocks, except that the testing blocks no longer provided feedbacks. The temporal structures of stimuli presentation in testing blocks are shown in Figure 4. Each single-task trial contained a stimulus for vehicle detection and an ISI varying between 1.25 and 1.5 s. Each dual-task trial contained a stimulus for vehicle detection followed by a dual-task module. In the dual-task, one-third trials randomly entered the dual-task module from entrance-1 and went through a black crosshair ISI (1.25–1.5 s), a red crosshair (1 s), a scene similarity judgment question, and another black crosshair ISI (1.25–1.5 s). The other two-third trials entered the dual-task module from entrance-2, which just contained a black crosshair ISI (1.25–1.5 s).

The whole experiment was divided into two sessions, each with one 40-trials single-task practicing block, three 200-trials single-task testing blocks, one 40-trials dual-task practicing block, and five 120-trials dual-task testing blocks. A single-task or dual-task practicing block consisted of all four stimulus types, each with 10 trials, and all trials were presented randomly. The mean duration of a single-task block was 418.0 s, and the mean duration of a dual-task block was 392.8 s. The stimuli in the testing set were first equally split to the two tasks by type, and then equally split to the two sessions by type. Thereafter, the testing stimuli were randomly distributed to three blocks (single-task) or five blocks (dual-task), respectively. The order of all the randomizations was different for each participant. The two experimental sessions for the same participant were performed over 2 days in order to keep the participants in good spirits during the experiment. In the first session, participants performed the single-task first, and in the second session they performed the dual-task first.

### 2.4. Behavioral Data Recording and Analysis

In the experiment, the participants used two keys (F and J) for behavioral feedback on both vehicle detection and scene similarity judgment tasks, with key F pressed with the left hand and key J pressed with the right hand. In the vehicle detection task, 25 participants (13 males, 12 females, named Group A) were asked to press F when a vehicle was detected and J when no vehicle was detected, while the other 25 participants (13 males, 12 females, named Group B) were asked to press the opposite key. In the scene similarity judgment task, participants were asked to press the key on the same side of the candidate image that they perceived as more similar to the previous stimulus.

During the experiment, the participants were required to stay focused and perform the task quickly and accurately, and were asked to take a brief break (about 3 min) after each block to keep them alert for the tasks. Participants' keystrokes for vehicle detection, keystrokes for scene similarity judgment, and the reaction time (RT) were recorded, and their vehicle detection and scene similarity judgment accuracies were calculated. The difference between participants' behavioral data under each experimental condition (i.e., simple-scene vs. complex-scene, and single-task vs. dual-task) were examined using two-sample *t*-test (two-tailed).

### 2.5. EEG Recording and Preprocessing

Participants were fitted with an ActiCHamp EEG system supplied by the Brain Products Company. Sixty-four channels, including one reference channel (channel Iz) and sixty-three EEG channels, were positioned on the head according to the international standard 10-10 system. The EEG sampling rate was 1,000 Hz and the impedance of each channel was kept below 10 kΩ prior to the beginning of recording.

Preprocessing of the EEG recording of each participant was conducted offline using the EEGlab toolbox (Delorme and Makeig, 2004) with MATLAB (version 18a). First, the continuous EEG data were down sampled to 250 Hz and applied 0.5–100 Hz band-pass and 50 Hz notch filters. Second, the data were re-referenced to the average of all scalp electrodes. Third, the data were segmented into 1,200 ms epochs (200 ms before and 1,000 ms after trial onset). Improbable epochs were discarded using the probability test (parameters: 6 SD for individual electrode channels, 2 SD for all electrode channels; Smout et al., 2019), and about 5.0% of the epochs were removed. We then conducted independent components analysis (ICA) on the remaining epochs and identified components representing blinks, saccades, and muscle artifacts with the help of the SASICA plugin (Chaumon et al., 2015) for EEGlab. On an individual average, about 25.0% of the independent components were removed as artifacts. Data from two participants in Group A with too many artifacts were discarded completely, so the total number of participants for the follow-up EEG analysis was 48. Finally, the artifact-free epochs were aligned to the stimulus onset and baseline corrected based on the mean response from 0 to 200 ms prior to each stimulus onset.

The subsequent ERP analysis was conducted based on epochs with correct behavioral feedbacks. The EEG epochs were sorted into the following experimental bins depending on the presence or absence of target, scene complexity, and task type: 2 target conditions (target and non-target) ×2 scene conditions (simple-scene and complex scene) ×2 task types (single-task and dual-task).

### 2.6. ERP Analysis

**Figure 5:**
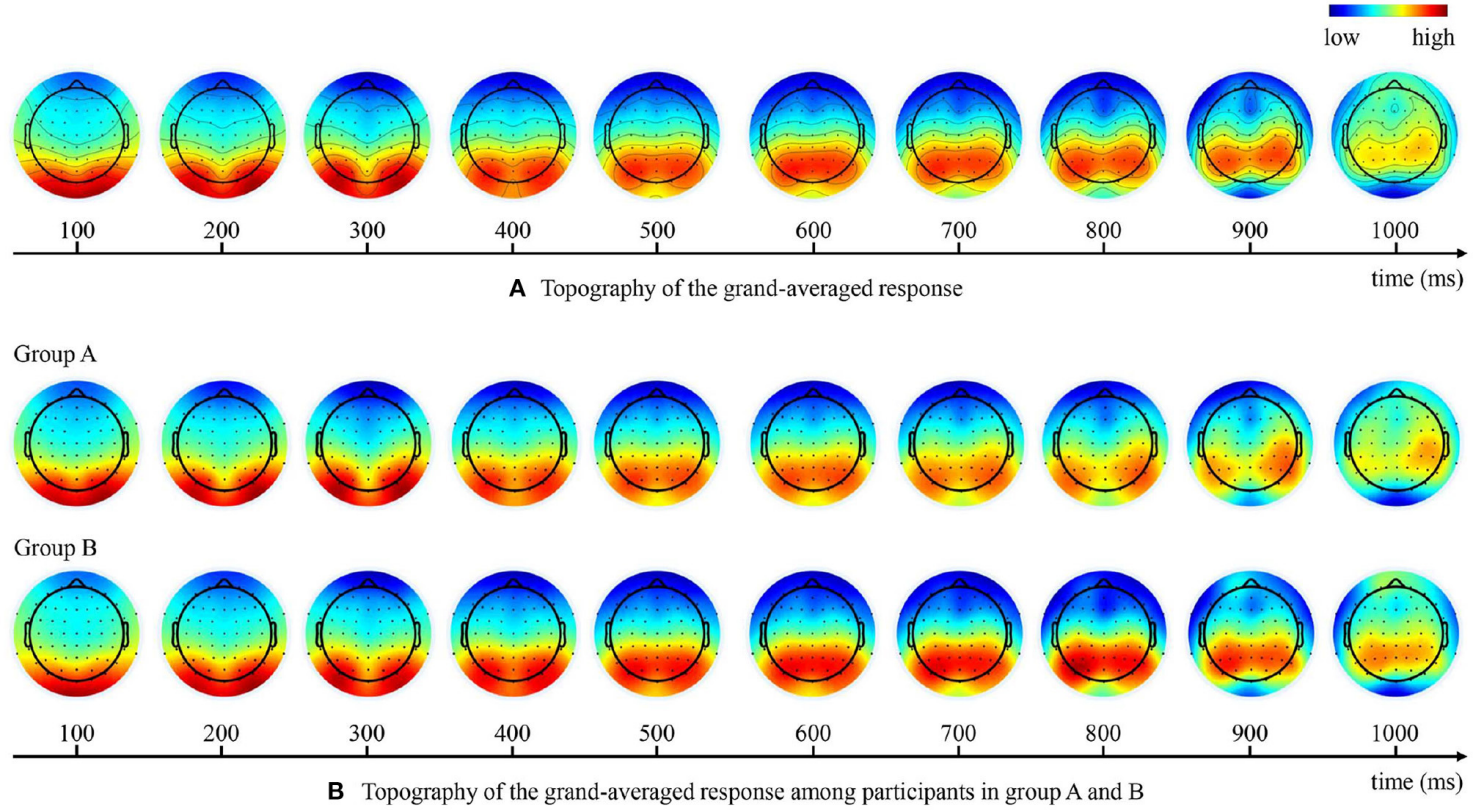
Topography of the grand-averaged EEG response at different time points. **(A)** Brain response topographies of all participants. **(B)** Brain response topographies among participants in group A and B.

The ERP analysis was carried out in the occipital and centro-parietal brain regions that showed strong EEG responses during the experiment, as shown in Figure 5A. Specifically, two clusters of electrodes were selected: six occipital EEG electrodes (O1, O2, PO3, PO4, PO7, PO8) and five central centro-parietal EEG electrodes (Cz, CPz, Pz, CP1, CP2). Epochs in each bin were averaged within individual participant to produce the ERPs of the two clusters of electrodes. It is worth noting that we have examined the ERPs from bilateral centro-parietal and parietal electrodes (CP3, CP4, CP5, CP6, P1, P2, P3, P4, P5, P6) that also showed strong EEG responses. The ERP waveforms in the bilateral centro-parietal and parietal electrodes were somewhat similar to those of the central centro-parietal electrodes but with less significant differences between experimental conditions. Therefore, the subsequent ERP analysis were conducted using the central centro-parietal electrodes (Cz, CPz, Pz, CP1, CP2). The choice of the central centro-parietal EEG electrodes in this study was consistent with that of previous ERP study on visual target detection (Luo and Ding, 2020).

For inter-condition ERP comparison, two-tailed two-sample *t*-test was performed between experimental bins, with a controlled false discovery rate (FDR) *p* < 0.05. We confirmed that the data came from the normal distributions with equal variance before conducting the two-sample *t*-test. In order to examine the difference between the latencies of brain response, the Jackknife approach (Miller et al., 1998) was adopted that iteratively removed one participant and computed the peak latency averaged over the remaining participants. The significance level was then computed on the basis of the mean peak latency across all participants and the standard deviation of the latencies obtained using the Jackknife approach (Luo and Ding, 2020).

### 2.7. ERP Analysis on Participants With High and Low Performance

To investigate the relationship between ERP and participants' behavioral performance, we divided the participants into high and low performance groups based on their reaction time in vehicle detection or accuracy in scene similarity judgment. For the grouping of participants based on reaction time, between-group differences were analyzed using single-task and dual-task ERPs, and for the grouping of participants based on accuracy in scene similarity judgment, between-group differences were analyzed using dual-task ERPs. For each condition, sixteen high/low performers were selected for comparison because the remaining participants showed relatively small differences. We also tested the choice of 12 high/low performers and found no significant change in the results of the ERP analysis.

When reaction time in vehicle detection was used as the performance indicator, the short RT group (RT = 0.729 ± 0.080 s, mean ± SD) and long RT group (RT = 1.147 ± 0.133 s, mean ± SD) were significantly differed in RT (*p* < 0.001, two-sample *t*-test) and had no significant difference in vehicle detection accuracy (short RT group vs. long RT group = 0.983 ± 0.008 vs. 0.986 ± 0.008, mean ± SD, *p* = 0.146, two-sample *t*-test).

When accuracy in scene similarity judgment was used as the performance indicator, the high accuracy group (accuracy = 0.925 ± 0.022, mean ± SD) and low accuracy group (accuracy = 0.774 ± 0.041, mean ± SD) were significantly differed in scene similarity judgment accuracy (*p* < 0.001, two-sample *t*-test) and had no significant difference in time spend on judgements (high accuracy group vs. low accuracy group = 1.587 ± 0.535 s vs. 1.433 ± 0.252 s, mean ± SD, *p* = 0.320, two-sample *t*-test).

## 3. Results

### 3.1. Results of Behavioral Test

Participants' vehicle detection accuracy, vehicle detection RT, and scene similarity judgment accuracy are shown in Table 2. In both single-task and dual-task, participants' RT was significantly longer (*p* < 0.001, two-sample *t*-test) and vehicle detection accuracy was significantly lower (*p* < 0.001, two-sample *t*-test) in complex-scene than that in simple-scene, which suggested that the high complexity of visual scene interferes with object detection. Furthermore, for simple-scene stimuli, when the dual-task was engaged, participants' vehicle detection accuracy was not significantly changed (*p* = 0.279, two-sample *t*-test), but their RT was significantly longer (*p* < 0.001, two-sample *t*-test) than that in the single-task, which indicated that attention to the background details of simple-scene reduced the speed but didn't influenced the accuracy for target detection. For complex-scene stimuli in the dual-task, participants' vehicle detection accuracy was higher (*p* = 0.004, two-sample *t*-test) and RT was significantly longer (*p* < 0.001, two-sample *t*-test) than that in the single-task, which demonstrated that attention to the background details slowed down the speed but improved the accuracy of target detection in complex scenes. The accuracy of scene similarity judgments for simple-scene and complex-scene didn't have significant difference (*p* = 0.748, two-sample *t*-test), which indicated that the difficulty of scene similarity judgments was well-balanced between the two groups of scene complexity and would not introduce a biased factor into participants' vehicle detection. It is worth noting that only one-third of the testing stimuli in the dual-task were followed by scene similarity judgement questions, which inevitably led participants to selectively observe the background of a portion of the stimuli. Although this issue was inevitable, it did not have a significant impact on the current study because the questions arose randomly, as evidenced by a significantly higher correction rate of participants' scene similarity judgment (85.5%) than random (i.e., 50%). In addition, there was no group-wise differences in RT or vehicle detection accuracy between genders or keystroke hands (*p* > 0.05, two-sample *t*-test).

**Table 2:** Results of behavioral test.

		**Simple scene**	**Complex scene**	***p*-value**
RT(s)	Single-task	0.599 ± 0.092	0.811 ± 0.190	<0.001
	Dual task	0.998 ± 0.344	1.267 ± 0.446	<0.001
	*p*-value	<0.001	<0.001	
Acc-VD	Single-task	0.992 ± 0.009	0.970 ± 0.014	<0.001
	Dual task	0.990 ± 0.010	0.978 ± 0.012	<0.001
	*p*-value	0.279	0.004	
Acc-SJ		0.857 ± 0.067	0.853 ± 0.077	0.748

*RT, reaction time; Acc-VD, vehicle detection accuracy; Acc-SJ, scene similarity judgment correction rate. Values in the table are expressed as mean ± SD. The p-values were obtained with two-tailed two-sample t-test*.

Taking above behavioral results together, both the scene complexity and task-relevance of background had significant effects on participants' target detection performance at group level.

### 3.2. Results of ERP Analysis

As shown in the topography of the grand-averaged responses in Figure 5A, the strongest brain response initially concentrated in the occipital regions and then shift to the centro-parietal regions over time. As the participants were asked to respond with a keystroke to report their detection results, the late ERP components may also reflect other neural processes such as the movement-related potentials (Kok, 1986). However, we believe that our results were not dominated by motor-related responses because we have eliminated the effects of lateralized signals on the holistic results by experimental design. In our experiments, participants in the same group responded less to target with one hand and more to non-target with the other hand, so that the motion-related potentials within one participant group were lateralized approximately 700 ms after stimulus onset, as shown in Figure 5B. Nevertheless, since we divided the subjects into two groups using opposite hands for the keystroke response, the topography of the grand-averaged responses at different time points (Figure 5A) showed no obvious laterality holistically.

**Figure 6:**
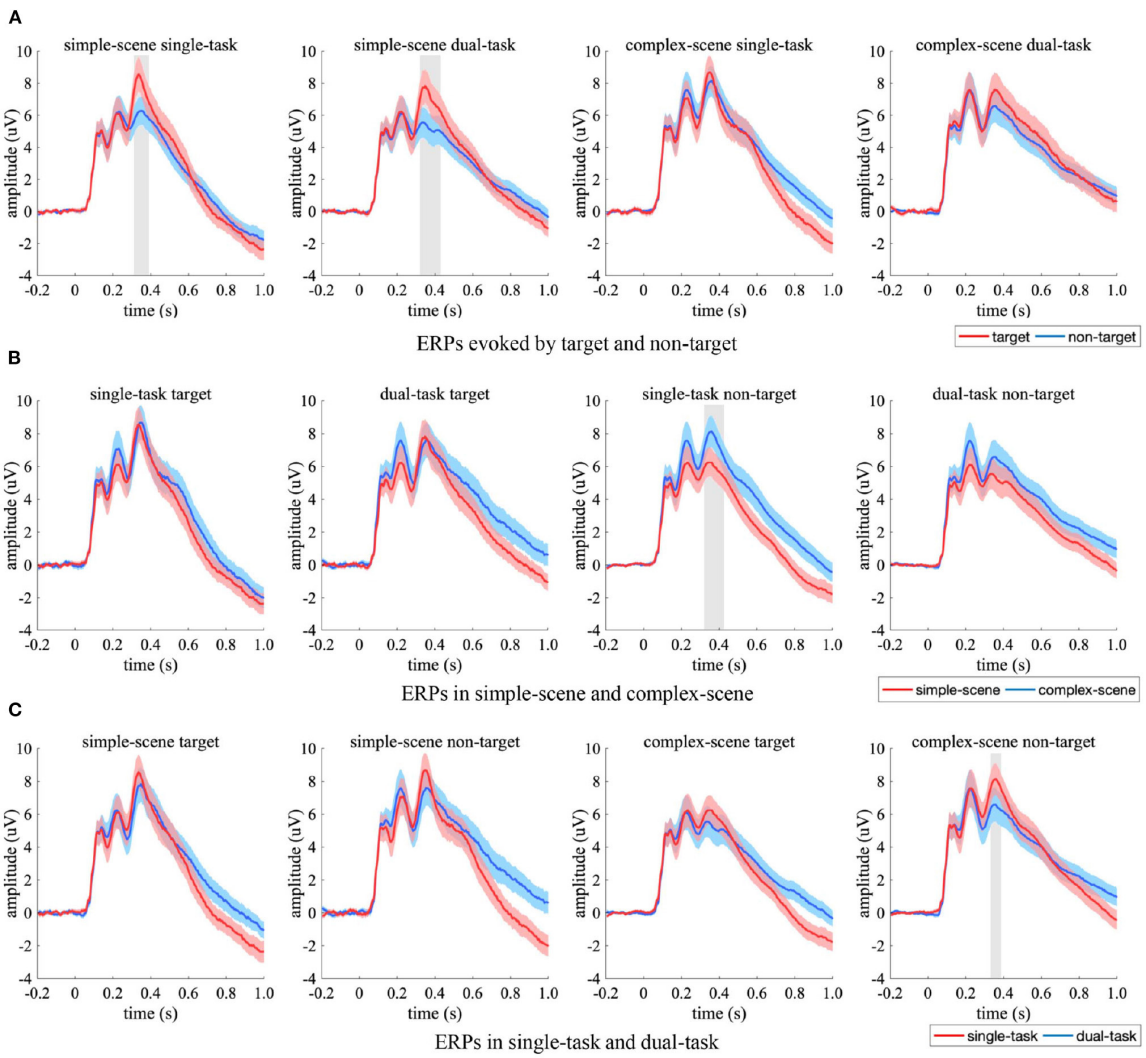
Comparison of ERPs in the occipital EEG electrodes. **(A)** ERPs evoked by target (red) and non-target (blue), **(B)** ERPs in simple-scene (red) and complex-scene (blue), **(C)** ERPs in single-task (red) and dual-task (blue). The shaded area indicates 95% confidence interval. Shaded gray regions denote the time intervals where there are significant differences between the two conditions (*p* < 0.05, two-sample *t*-test, FDR corrected).

The ERP from occipital EEG electrodes were compared between the experimental conditions (Figure 6). Significant ERP differences between experimental conditions occurred in amplitude rather than latency. Specifically, the target-evoked ERPs had significant stronger amplitude than non-target-evoked ones in simple-scene (Figure 6A, 304–372 ms in single-task, 320–420 ms in dual-task, *p* < 0.05, two-sample *t*-test, FDR corrected), which indicated that targets and non-targets evoked occipital responses of varying strength when scene complexity was simple. When there was no target in the stimuli, the ERPs corresponding to complex-scene showed overall higher amplitude than that corresponding to simple-scene (Figure 6B), which was significant in single-task (328–424 ms, *p* < 0.05, two-sample *t*-test, FDR corrected). This result indicated that higher background complexity elicited stronger occipital response especially when participants' attention was focused. The ERPs in the single-task had an overall stronger peak amplitude than those in the dual-task (Figure 6C), which was significant for non-target stimuli in complex-scenes (344–396 ms, *p* < 0.05, two-sample *t*-test, FDR corrected). This result implied that attention distraction may weaken the occipital response in target detection especially when distractors were complex.

**Figure 7:**
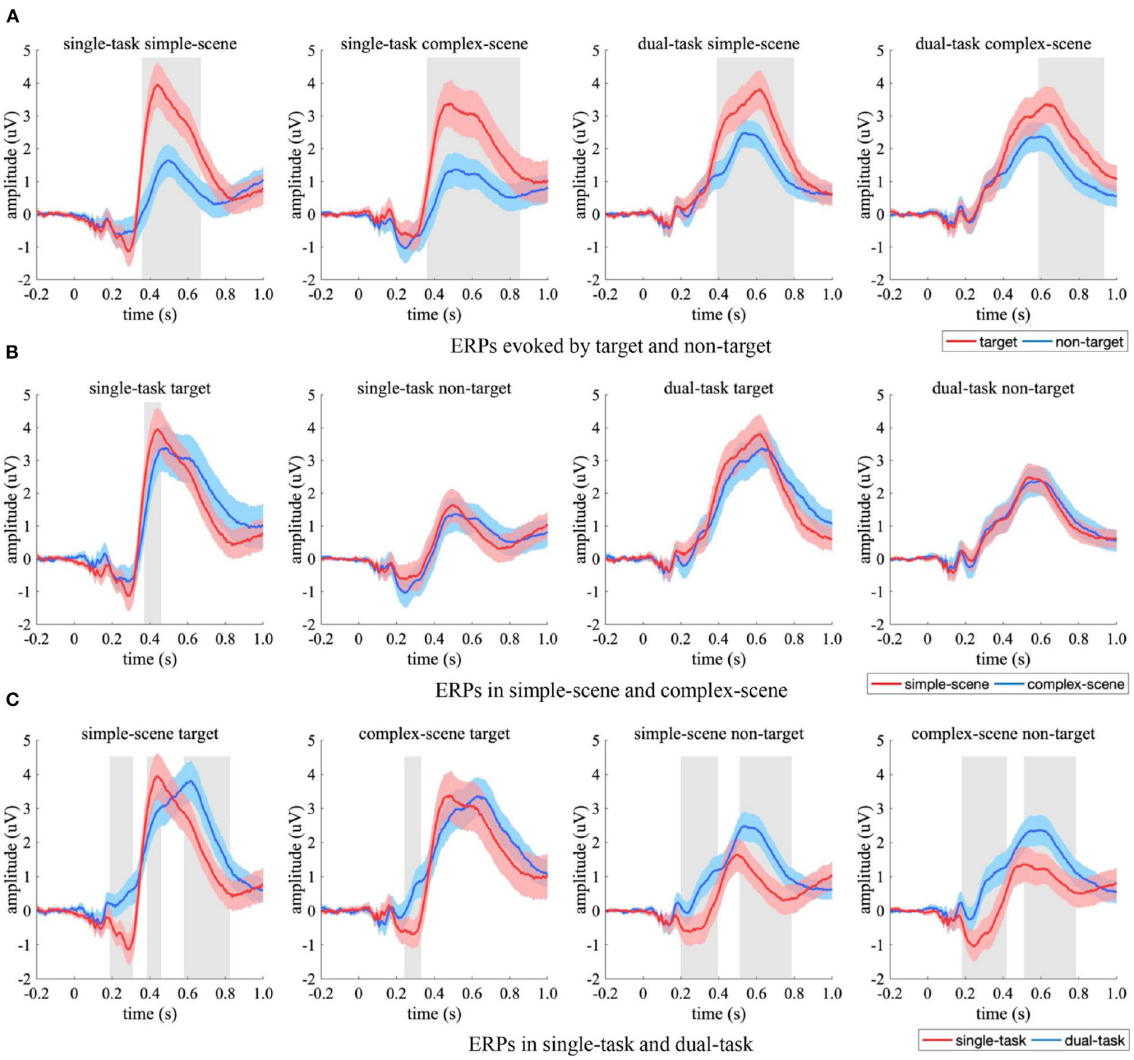
Comparison of ERPs in the centro-parietal EEG electrodes. **(A)** ERPs evoked by target (red) and non-target (blue), **(B)** ERPs in simple-scene (red) and complex-scene (blue), **(C)** ERPs in single-task (red) and dual-task (blue). The shaded area indicates 95% confidence interval. Shaded gray regions denote the time intervals when there are significant differences between the two conditions (*p* < 0.05, two-sample *t*-test, FDR corrected).

The ERPs from centro-parietal EEG electrodes were compared between experimental conditions (Figure 7). Target-evoked ERPs had overall significant stronger amplitudes than non-target-evoked ones (Figure 7A, *p* < 0.05, two-sample *t*-test, FDR corrected), which may be due to an imbalanced effect of the samples that made targets and non-targets evoked brain responses of varying strength in the centro-parietal regions, especially when participants' attention was focused (i.e., in the single-task). The ERPs in simple-scene had significantly stronger peak amplitude for target in the time interval of 356–436 ms (Figure 7B, *p* < 0.05, two-sample *t*-test, FDR corrected) than that in complex-scene, and had similar amplitudes under other experimental conditions (*p* > 0.05, two-sample *t*-test, FDR corrected). Moreover, the ERPs in simple-scene showed overall shorter peak latency than that in complex-scene (simple scene vs. complex scene = 520.0 ± 26.6 ms vs. 608.0 ± 19.5 ms, Mean ± SD, *p* < 0.001, two-sample jackknife procedure), which implied that an increase in background complexity may elongate the peak latency of centro-parietal response. The peak latency of ERPs in the dual-task was significant longer than that in the single-task (*p* < 0.001, two-sample Jackknife test), and the amplitude evoked by targets was similar between tasks (*p* > 0.05, two-sample *t*-test, FDR corrected) while that evoked by non-targets was significant stronger in the dual-task (*p* < 0.05, two-sample *t*-test, FDR corrected) (Figure 7C). These results indicated that attention distraction could cause a significant time delay in the centro-parietal responses, and active observation for background details could increase the amplitude of the centro-parietal responses to non-target stimuli.

### 3.3. Results of ERP Analysis on Participants With High and Low Performance

**Figure 8:**
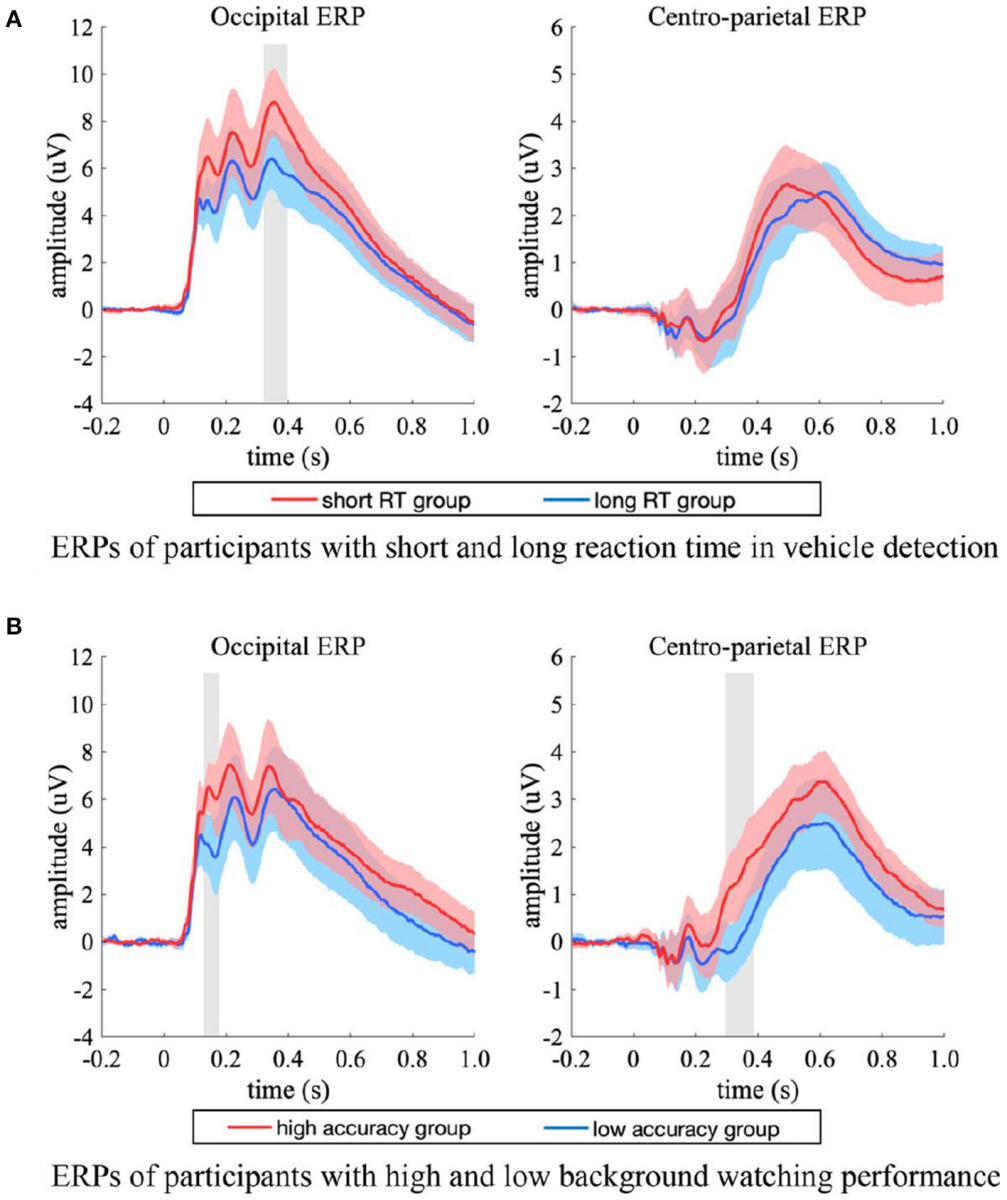
ERP differences between participants with high and low performance. **(A)** The occipital (left) and centro-parietal (right) ERPs of short (red) and long (blue) RT groups, the ERPs were from both the single-task and the dual-task. **(B)** The occipital (left) and centro-parietal (right) ERPs of high (red) and low (blue) accuracy groups, the ERPs were from the dual-task. The shaded area indicates 95% confidence interval. Shaded gray regions denote the time intervals when there were significant differences between the two groups (*p* < 0.05, two-sample *t*-test, FDR corrected).

For the short and long RT groups, the ERP in the occipital electrodes differed in a time interval of 340–388 ms (Figure 8A, left). The two groups had comparable peak latency (*p* = 0.159, two-sample jackknife procedure), while the short RT group showed significant stronger peak amplitude than that of the long RT group (*p* < 0.05, two-sample *t*-test, FDR corrected). For the ERPs in the centro-parietal electrodes (Figure 8A, right), the two groups had comparable peak amplitude (*p* = 0.960, two-sample *t*-test), while the short RT group showed significant shorter latency (short RT group vs. long RT group = 500.0 ± 13.1 ms vs. 620.0 ± 15.9 ms, mean ± SD, *p* < 0.001, two-sample jackknife procedure). These results indicated that the amplitude of occipital response was positively related with participants' reaction speed in target detection, while the latency of centro-parietal response was positively related with participants' reaction time.

For the high and low accuracy groups, significant ERP differences between experimental conditions occurred in amplitude rather than latency. The ERPs in the occipital electrodes differed in a time interval of about 100–400 ms (Figure 8B, left), and the ERPs in the centro-parietal electrodes differed after 300 ms post stimulus onset (Figure 8B, right). The high accuracy group had higher amplitude in both occipital and centro-parietal ERPs. Specifically, the high accuracy group had significant stronger occipital response amplitude in the time interval of 132–164 ms (*p* < 0.05, two-sample *t*-test, FDR corrected), and had significant stronger centro-parietal response amplitude in the time interval of 296–396 ms (*p* < 0.05, two-sample *t*-test, FDR corrected), which implied that the amplitude of EEG response was positively related with participants' background observation performance.

## 4. Discussion

How the human brain processes natural scenes is a fundamental question (Vinje and Gallant, 2000). Previous studies investigated the neural mechanisms of visual processing using synthetic or curated stimuli (Davenport and Potter, 2004; Torralba, 2009; Sun et al., 2011; Prass et al., 2013; Barenholtz, 2014; Hagen and Laeng, 2016; Smout et al., 2019; Luo and Ding, 2020). We suggest that studying the mechanism of visual processing with objects in their native backgrounds would better capture the essence of the real-world problem. The current study constructed a new stimulus set with two levels of scene complexity and guided participants actively or passively attend to the background details in the vehicle detection task. Results from behavioral tests and ERP analysis revealed the effects of scene complexity and task-relevance of background on participants' response time, accuracy and brain response in vehicle detection. We suggest that these phenomena may be attributed to the attentional cueing and sensory evidence accumulation effects of native backgrounds.

### 4.1. Native Background and Visual Object Processing

Effective processing for visual objects relies on the neural encoding of both target and background (Luo and Ding, 2020). The visual background may contain task-relevant contextual information and task-irrelevant distractors, and has a significant impact on participant's performance (Naber et al., 2012). Concretely, context is a statistical property of the world that provides critical information to facilitate faster and more accurate resolution of perceptual reasoning tasks (Mottaghi et al., 2014). Distractors, on another note, are task-irrelevant information that may modulate cortical responses and interfere with task performance (Cave and Chen, 2016; Itthipuripat et al., 2019). Extensive body of studies have demonstrated the positive effects of context in perceptual tasks such as object detection, object recognition, and object segmentation. Specifically, context is an effective cue for human to detect objects, especially when the appearance information of object is impoverished (Parikh et al., 2012). Moreover, contextual information can modulate the allocation of human attention and thus promote the process of recognizing objects embedded in the scene (Sun et al., 2011). In addition, contextual information can facilitate reliable execution of difficult tasks such as object segmentation at very low resolutions (Torralba, 2009). On the other hand, signals from visual distractors inevitably leak through selective filters and compete with the target for processing resources (Cave and Chen, 2016), which could reduce task performance in object recognition (Davenport and Potter, 2004), categorization (Prass et al., 2013), and detection (Luo and Ding, 2020).

Previous studies of visual processing have investigated the role of context and distractors independently in the absence of a stimulus set with objects in their native backgrounds. They typically embedded objects in different scenes that were considered distractors to visual object processing (Davenport and Potter, 2004; Prass et al., 2013; Luo and Ding, 2020) and reached the conclusion that it was more difficult to identify or detect objects in complex scenes. Alternatively, human performance was compared between conditions that objects were in their native and un-native backgrounds (Sun et al., 2011) or in different resolutions (Torralba, 2009; Barenholtz, 2014), concluding that contextual information facilitated visual target processing. However, context and distractors could be intermingled in the realistic scenes and might attach to the same content with conflicting effects on visual object processing. Taking the images shown in Figure 2 as an example, the vehicles may appear in the roads but not on the roofs. The road directs attention to find vehicles along the road, while the building guides attention to search for vehicles in non-building areas (e.g., open spaces near buildings). In this regard, both roads and buildings exert positive incentives for attention shifting. However, the salient contents in the background (e.g., the buildings) may also automatically attract attentions (Lavie, 2005; Beste et al., 2011) and become a distraction that interferes with task performance. Using stimuli where the targets are in their native backgrounds helps us to understand more clearly the mechanism of visual object processing in realistic scenes.

Previous studies have observed that the targets and distractors mediate attention in visual object processing through biased-competition processes (Duncan et al., 1997), which are modulated by stimulus saliency and task intention (Knudsen, 2007), and can be achieved by enhancing target features (Mazza et al., 2009) or suppressing distractors (Hopf et al., 2006). It has been corroborated that proactive top-down processes are critical in preventing bottom-up attention from being captured by salient distractors (Mueller et al., 2009). This is supported by the results of the current study. In the single-task experiment, participants were asked to focus on the vehicle detection task, which allowed them to resist the influence of irrelevant background information on the detection task, as reflected in faster reaction speed and higher accuracy than in the dual-task experiment that required them to observe detailed background information (Table 2). In addition, we observed some differences in occipital and centro-parietal ERPs related to scene complexity and attentional state to the background, which may be related to the variations in attentional cueing and sensory evidence accumulation effects across experimental conditions. These issues are discussed in detail in the following sections.

### 4.2. Attentional Cueing Effect of Native Backgrounds in Vehicle Detection

Attention is the basis of visual processing, which is modulated by both stimulus-driven and goal-directed factors (Yantis, 2000). It has been demonstrated that scene context facilitated the processing of visual objects by providing attentional cues, which could increase the apparent contrast of visual stimuli, enhance early neurophysiological responses in visual cortex, and elicit behavioral improvements such as increasing the speed and accuracy of visual processing (Stormer et al., 2009; Itthipuripat et al., 2014). In current study, we observed a late occipital positivity (LOP) peaked approximately 350 ms after stimulus onset (Figure 6), which has been regarded as a cue-induced activation of the visual cortex that would lead to more efficient perceptual processing of visual information at the cued location (Feng et al., 2014), and has been found to associated with the rapid covert shift of spatial attention in visual search (Simpson et al., 2011). As a neural representation for the cue-directed attention, the amplitude of LOP reflected the attention-cueing effects across experimental conditions in this study.

**Figure 9:**
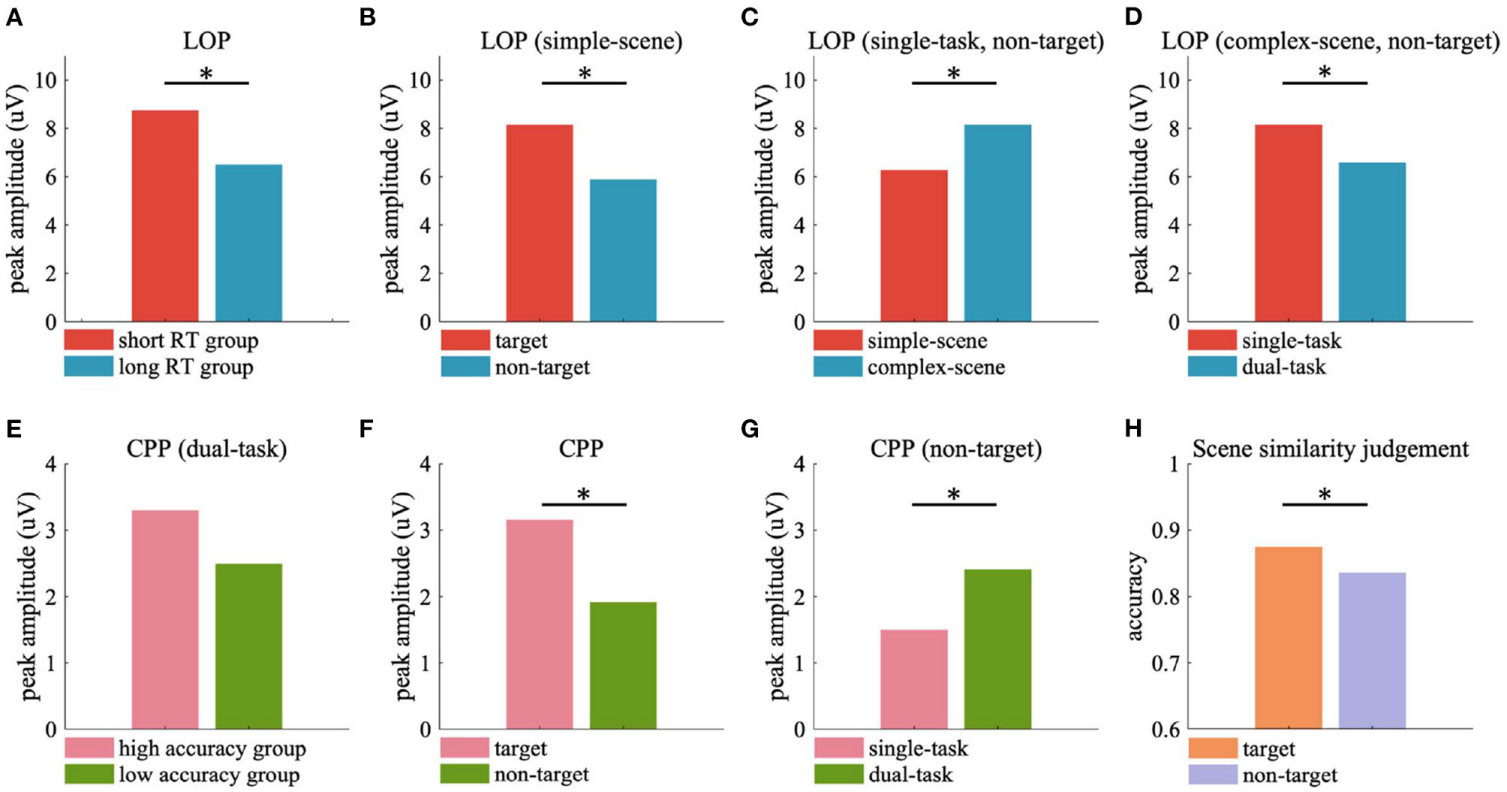
Peak ERP responses and scene similarity judgement accuracy averaged over participants. **(A)** Peak LOP amplitude of short RT and long RT groups. **(B–D)** Peak LOP amplitude evoked by target and non-target in simple-scene **(B)**, simple-scene and complex-scene without target in single-task **(C)**, single-task and dual-task in complex-scene without target **(D)**. **(E)** Peak CPP amplitude of high accuracy and low accuracy groups. **(F)** Peak CPP amplitude evoked by target and non-target. **(G)** Peak CPP amplitude evoked by non-target in single-task and dual-task. **(H)** Accuracy of scene similarity judgement for stimuli with target and without target. Significant differences are indicated by black stars (*p* < 0.05, two-sample *t*-test).

The current results of ERP analysis showed that the amplitude of LOP in the participant group with short RT was significantly stronger than that with long RT (Figure 9A), which implied that the attentional cueing effect overall positively related with participants' task performance in object detection. Moreover, we observed stronger LOP when attention was aroused by salient visual content such as vehicle targets (Figure 9B) or complex background contents (Figure 9C), or when attention was focused due to the task requirement (i.e., in the single-task, Figure 9D). In these cases, the presence of vehicle target and the concentration of attention on object detection task were accompanied by better behavioral performance, while higher scene complexity was accompanied by poorer behavioral performance (Table 2). These results demonstrated that the attentional cueing effects were influenced by both stimulus-driven and psychological factors, which suggest that the LOP amplitude reflects the extent to which attention is cued, rather than the contribution of attention to the task. High performance in target detection tasks also relies on the suppression of excessive attentional cues.

### 4.3. Sensory Evidence Accumulation Effect of Native Backgrounds in Vehicle Detection

We observed that in the vehicle detection task, a centro-parietal positivity (CPP) reached a maximum between 400 and 650 ms after stimulus onset (Figure 7). The CPP is a late ERP component specifically linked to perceptual decision-making (Kelly and O'Connell, 2015; Herding et al., 2019), which has strong resemblance to the classical P300 (O'Connell et al., 2012) and is suggested to be a correlate of the accumulation of sensory evidence (Kelly and O'Connell, 2013; Loughnance et al., 2016; Tagliabue et al., 2019). Previous studies have shown that the timing of CPP varies closely with participant's reaction time (Kutas et al., 1977), while its amplitude is strongly related with the subjective experience of stimulus clarity, which positively correlates with the level of visual awareness rating (Tagliabue et al., 2019). Moreover, the amplitude of CPP is subject to additive effects of directed attention and motivated attention for visual object processing, which are induced by bottom-up saliency and top-down task-relevance, respectively. Specifically, salient targets evoke the largest CPP and non-salient backgrounds evoke the least CPP (Ferrari et al., 2008).

In the dual-task of current study, the participants with high background observing performance showed stronger CPP amplitude than those with low performance (Figure 9E), which implied that participants with higher scene similarity judgement accuracy may accumulate more useful sensory evidences from the background scenes. What's more, target stimuli were observed to evoke stronger CPP than non-target stimuli (Figure 9F), which may be the contribution of motivated attention induced by the relevance of task requirement of detecting vehicles. The CPP evoked by non-targets in the dual-task was stronger than that evoked by non-targets in the single-task (Figure 9G), which may reflect the increased demand for background-derived sensory evidence for additional scene observing requirement. The CPP evoked by tarets in both single-task and dual-task had similar amplitude (Figure 7C, left two sub-figures), but participants showed significant higher accuracy in scene similarity judgment for target stimuli than non-target stimuli (Figure 9H, target vs. non-target = 0.875 ± 0.067 vs. 0.836 ± 0.092, *p* < 0.05, two-sample *t*-test), with similar time spend on judgement (target vs. non-target = 1.603 ± 0.474 vs. 1.540 ± 0.446 s, *p* = 0.498, two-sample *t*-test). These findings were consistent with the conclusion of previous study that scene recognition could be facilitated by the presence of consistent objects (Mack and Palmeri, 2010). The presence of semantically consistent objects facilitated the capture of core features of the scene, allowing the participants to accumulate the sensory evidence more effectively when there was an additional demand to actively observing the background details, and to perform better in recalling the scene features in the following scene similarity judgement task.

### 4.4. General Discussion

The achievement of meaningful contextual processing relies on the separation of target and background (Vanmarcke et al., 2016). Complex native scenes may provide more attention-guiding cues, but salient scene contents also increases the difficulty to target-background separation, so its negative effect on target processing may overwhelm the positive effect of incremented semantic cues. In this study, participants' behavioral and neural differences in the single-task and dual-task demonstrated that object detection in natural scenes does not require the utilization of detailed contextual information, and brief attentional cues are sufficient for participants to efficiently localize the visual targets. This is consistent with previous findings that simple contextual information can be very helpful for visual object processing, for example, contextual information in blurred scenes can make objects more recognizable (Barenholtz, 2014), which is particularly beneficial for visual processing of objects at low resolution (Torralba, 2009). The above results show that complex scenes may provide more cues for attention-shifting, but may also distract attention from object detection, thus slowing the accumulation of sensory evidence for decision-making. Active attention to background content enables participants to obtain more detailed scene information but spreads their attention from object detection, thereby reducing the effect of attentional cues and slowing the perceptual evidence accumulation for decision-making. Our findings imply that object processing is inextricably linked to the processing of background scene information, which has clear task-relevance (attend or unattended) and significantly affects participant's object detection performance. While contextual information is essential, excessive attentional cues and fine-grained but semantically irrelevant scene information do not seem to benefit real-world object detection.

### 4.5. Limitations

We suggest that several limitations should be taken into account when interpreting the findings of the current study. First, the sample size of stimulus set in this study was relatively small. In order to avoid as much as possible the impact of predictable confounding factors on the experiment, we cropped stimulus patches from a single dataset to ensure that they were obtained under the same imaging conditions and screened these patches qualitatively and quantitatively, resulting in a relatively small number of patches used for the experiment. We advocate further studies using larger stimulus set to replicate the results of the current study. Second, since the participants may adopt multiple strategies when conducting the experimental tasks, the influence of different task execution strategies cannot be ruled out as possible explanations for the observed behavioral and neurological differences across experimental conditions. In further studies, more controllable experimental designs should be considered to test the current findings.

## 5. Conclusion

The current study explored the role of scene complexity and task-relevance of the native background in visual object detection using a new stimulus set and an attention-guiding strategy, and provided an answer to the question of how the native background affects visual object detection. Behavioral and neurological results demonstrated that both bottom-up and top-down factors influence visual object detection, with a moderate amount of attentional cues and focused task-relevant attention being beneficial, while excessive attentional cues and fine-grained but semantically irrelevant contextual information being detrimental to real-world object detection. The current study bridges the gap in understanding the role of native background in object detection and may advance our understanding for the neural mechanism of real-world visual processing. These findings corroborate that efficient visual processing of real-world objects may involve a competition process between contextual information and distractors that co-exist in the natural scenes, culminating in a visual processing system with high task performance and high energy efficiency.

## Data Availability Statement

The dataset that support the findings of this study are available from the corresponding author upon reasonable request.

## Ethics Statement

The studies involving human participants were reviewed and approved by Helsinki Declaration of 1975, as revised in 2000. The patients/participants provided their written informed consent to participate in this study.

## Author Contributions

JL and YW designed the experiments and wrote the manuscript. JL contributed significantly to the conception of the study. YW, JY, and ZY recruited the participants and collected the data. YW and SR analyzed the data. MD, CZ, and WZ helped perform the analysis with constructive discussions. All authors contributed to the article and approved the submitted version.

## Conflict of Interest

The authors declare that the research was conducted in the absence of any commercial or financial relationships that could be construed as a potential conflict of interest.
