# Supplementary material for: How Native Background Affects Human Performance in Real-World Visual Object Detection: An Event-Related Potential Study
Source: Front Neurosci. 2021 Apr 30;15:665084. doi: 10.3389/fnins.2021.665084 (PMC8119748; doi:10.3389/fnins.2021.665084)
Supplement: Supplementary file 1 [file Data_Sheet_1.PDF]

# Supplementary Material

## 1 RESULTS OF ERP ANALYSIS ON PARTICIPANTS WITH HIGH AND LOW PERFORMANCE

To investigate the relationship between ERP and participants' behavioral performance, we divided the participants into high and low performance groups based on their reaction time in vehicle detection or accuracy in scene similarity judgment, and analyzed the ERP differences between the two groups accordingly. We tested the choice of 12 high/low performers and found no significant change in the results of the ERP analysis and results were shown in Figure S1.

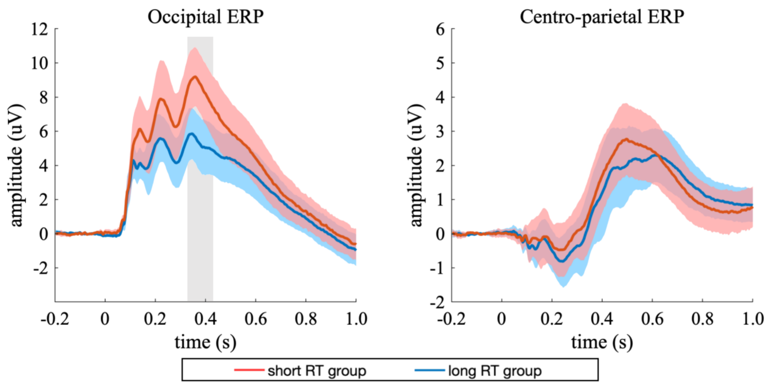


(a) ERPs of participants with short and long reaction time in vehicle detection

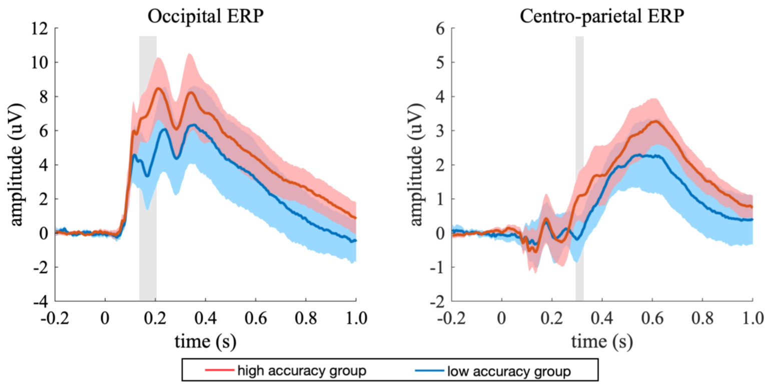


(b) ERPs of participants with high and low background watching performance

**Figure S1.** ERP differences between 12 high performance and 12 low performance participants. (a) The occipital (left) and centro-parietal (right) ERPs of short (red) and long (blue) RT groups. (b) The occipital (left) and centro-parietal (right) ERPs of high (red) and low (blue) accuracy groups. The shaded area indicates 95% confidence interval. Shaded gray regions denote the time intervals when there were significant differences between the two groups ($p<0.05$, two-sample $t$-test, FDR corrected).

When reaction time in vehicle detection was used as the performance indicator (Figure S1a), the short RT group (RT=0.701±0.072 seconds, mean±SD) and long RT group (RT=1.194±0.121 seconds, mean±SD) were significantly differed in RT ($p<0.001$, two-sample $t$-test) and had no significant difference in vehicle detection accuracy (short RT group vs. long RT group = 0.982±0.006 vs. 0.986±0.007, mean±SD, $p$=0.166, two-sample t-test). The short RT group showed significant stronger peak amplitude than that of

the long RT group in a time interval of 328-420 ms ($p<0.05$, two-sample $t$-test, FDR corrected) and shorter latency (short RT group vs. long RT group = 496.0 ±6.40 ms vs. 612.0±23.55 ms, mean±SD, $p<0.001$, two-sample jackknife procedure).

When accuracy in scene similarity judgment was used as the performance indicator (Figure S1b), the high accuracy group (accuracy=0.934±0.019, mean±SD) and low accuracy group (accuracy=0.758±0.033, mean±SD) were significantly differed in scene similarity judgment accuracy ($p<0.001$, two-sample $t$-test). The high accuracy group had significant stronger occipital response amplitude in the time interval of 140-204 ms and significant stronger centro-parietal response amplitude in the time interval of 296-316 ms ($p<0.05$, two-sample $t$-test, FDR corrected).
